# Totally Endoscopic Approach for TAVR Explantation and Subsequent Surgical AVR Using a 3D Endoscope System

**DOI:** 10.1016/j.jaccas.2025.104432

**Published:** 2025-07-30

**Authors:** Joon Young Kim, Duk-Woo Park, Jae Suk Yoo

**Affiliations:** a Department of Thoracic and Cardiovascular Surgery, Asan Medical Center, University of Ulsan College of Medicine, Seoul, Republic of Korea; b Department of Cardiology, Heart Institute, Asan Medical Center, University of Ulsan College of Medicine, Seoul, Republic of Korea

**Keywords:** 3-dimensional endoscope, AVR, minimally invasive cardiac surgery, TAVR explant, totally endoscopic cardiac surgery

## Abstract

**Objectives:**

Surgical aortic valve replacement (SAVR) after transcatheter aortic valve replacement (TAVR) has a higher mortality rate than standard SAVR. We present a successful minimally invasive TAVR explantation and SAVR using the lasso technique in conjunction with a 3D endoscope.

**Key Steps:**

1) Aortotomy was performed where the nitinol frame was easily palpable. 2) The first purse-string suture was placed around the edge of the frame. 3) After exposing the widest part of the frame, a second purse-string suture was placed at the same level. 4) The second lasso was used to expose the annulus, ensuring safe explantation.

**Potential Pitfalls:**

Comprehensive understanding of the prosthesis structure and the surgical dissection plane is essential to prevent aortic injury. Excessive traction can damage the annulus or aortic root.

**Take-Home Messages:**

Understanding the TAVR prosthesis and surgical plane simplified the explantation process. The lasso technique with a 3D endoscope facilitated explantation, even in high-risk patients.

## Graphical Abstract

**Figure:**
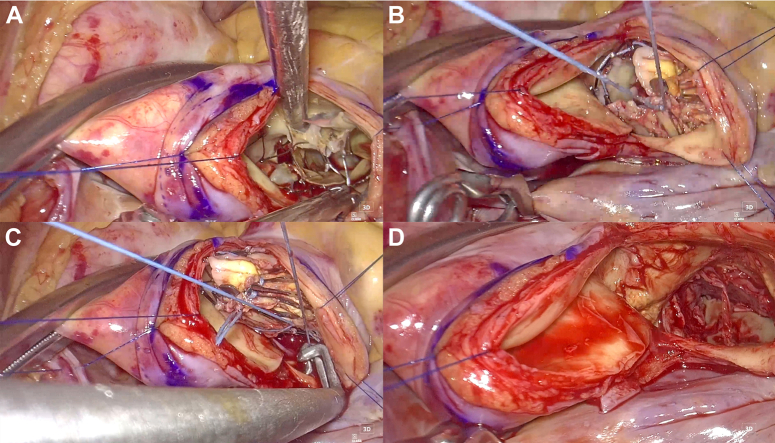

Transcatheter aortic valve replacement (TAVR) has emerged as a widely used alternative to surgical aortic valve replacement (SAVR) for the treatment of symptomatic aortic stenosis. The U.S. Food and Drug Administration has approved TAVR for all surgical risk categories. As TAVR procedures increase, so does the incidence of failed TAVR cases including structural valve degeneration, paravalvular leak, and infective endocarditis. However, owing to the considerable operative risk, only a few patients have undergone surgical intervention after a failed TAVR.^1^ The operative mortality rate for SAVR after TAVR has been reported at 14.1% and 13.6%, which is higher than the average for SAVR.^2^^,^^3^ One of the challenges in the surgical management of TAVR explantation is the adhesion of large stent frames to the ascending aorta and aortic root, complicating the procedure. Nevertheless, the lasso technique offers a simpler method for TAVR explantation.^4^ Despite advancements in minimally invasive cardiac surgery, cases involving TAVR explantation and aortic valve replacement (AVR) using minimally invasive techniques remain exceedingly rare. In this study, we present a successful case of TAVR explantation and SAVR utilizing the lasso technique through a minimally invasive 2-port, 3-dimensional totally endoscopic approach.

**Take-Home Messages**

• Understanding the TAVR prosthesis and the surgical plane simplifies the explantation process.
• The lasso technique, combined with a 3D endoscope, facilitates straightforward explantation, even in high-risk patients.

## Case Summary

An 85-year-old man who underwent TAVR with a CoreValve Evolut R 26 mm (Medtronic) in 2018 for severe degenerative aortic regurgitation (AR) presented with acute AR caused by right coronary cusp prolapse and severe dyspnea (NYHA functional class IV), necessitating immediate surgery. According to the Society of Thoracic Surgeons-American College of Cardiology Transcatheter Valve Therapy (STS-ACC TVT) Registry, the number of valve-in-valve TAVR procedures is steadily increasing, not only for aortic stenosis but also for AR.^5^ However, in South Korea, valve-in-valve TAVR for AR is not legally approved, making it unavailable. Consequently, TAVR explantation and AVR were planned.

## Procedural Steps

### Minimally invasive surgical approach and cardiopulmonary bypass setup

The patient was intubated using a double-lumen endotracheal tube and positioned at a 30° right-side-up tilt, with a cushion placed along the spine. A 3-cm thoracotomy was performed in the third intercostal space (ICS) along the right midclavicular line, and a 10-mm trocar was inserted into the fourth ICS at the anterior axillary line. A soft tissue retractor was positioned at the main thoracotomy incision. Cardiopulmonary bypass (CPB) was established percutaneously via the right femoral artery and vein. A left ventricular vent catheter was inserted into the right upper pulmonary vein through the thoracotomy in the third ICS. After the right atriotomy, a retrograde cardioplegia catheter was placed. The aorta was clamped using a detachable Glauber clamp (Cardiovision-Trytech), and retrograde del Nido cardioplegia was administered.

### The lasso technique

**Figure 1:**
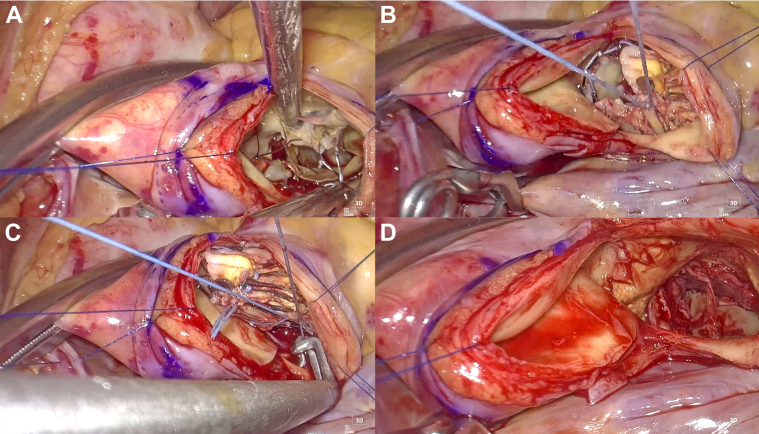
Intraoperative Images (A) Endarterectomy was performed between the frame and the aortic wall. (B) The first purse-string suture was placed at the distal edge, collapsing the distal segment. (C) The second purse-string suture was placed at the widest section, collapsing that area. (D) Image after the removal of the transcatheter aortic valve replacement prosthesis.

**Figure 2:**
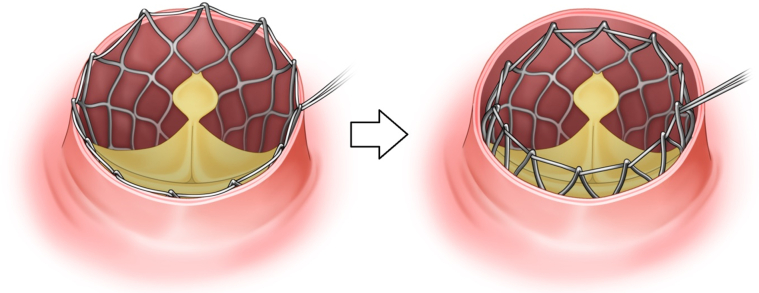
The First Step of the Lasso Technique for Explanting a Prosthesis With a Self-Expanding Frame A purse-string suture was placed at the distal edge of the frame and secured using a tourniquet or a tie.

**Figure 3:**
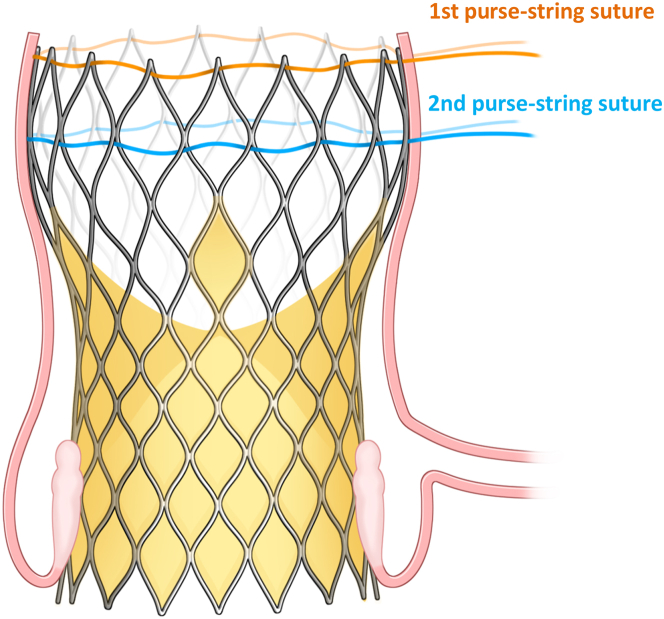
Structure of the CoreValve Evolut R and the Lasso Technique The first purse-string suture is placed at the distal edge of the frame, followed by a second purse-string suture at the widest part of the frame.

A transverse aortotomy was performed where the nitinol frame could be easily palpated with surgical instruments. The TAVR explantation was conducted using the lasso technique. A 2-0 polyester purse-string suture was placed around the edge of the frame and secured with a tie (Figure 1), creating space for an endarterectomy between the frame and the aortic wall. After exposing the widest part of the frame, a second 2-0 polyester purse-string suture was positioned and secured with a tie (Figures 2 and 3). Because the frame is narrowest at the waist, the second lasso quickly exposed the annulus level. Gentle traction was applied using the purse-string suture and tie, which acted as a valve holder, facilitating the separation of the prosthesis from the aortic valve despite tight adhesion.

After the explantation, the native valve was removed, and sutureless AVR was performed using a Perceval Plus Medium (Corcym). After the repair of the aortotomy, de-airing, and the repair of the right atriotomy, the aortic cross-clamp was released. The patient was successfully weaned off CPB without complications. The total CPB and aortic cross-clamp times were 113 minutes and 69 minutes, respectively.

## Outcome and Follow-Up

The patient was extubated 5 hours after surgery. Postoperative delirium delayed lung care, extending the intensive care unit stay to 4 days. On postoperative day 3, a transthoracic echocardiogram revealed satisfactory prosthetic valve function and no paravalvular leak. Although his ejection fraction decreased from 46% preoperatively to 37%, his overall condition improved, and the delirium subsided. The patient was discharged on postoperative day 9 without complications.

## Discussion

A recent study on TAVR explantation indicated that surgical mortality rates are high, and damage to surrounding structures is common.^6^ Notably, many surgeons perform these TAVR explant procedures without prior experience. Most patients who had TAVR initially had very high surgical risks, which may further elevate the risks associated with SAVR compared with their initial TAVR procedure. Both the nitinol frame and cobalt-chromium alloy TAVR prostheses are secured by radial force against the annulus and aortic root. This design can lead to embedding in the intima of the aortic wall, resulting in significant adhesions that complicate surgery. Attempting removal without specialized knowledge or experience could transform a straightforward SAVR into a complex root replacement because of complications such as root injury, aortic dissection, or left ventricular rupture. Therefore, it is imperative for surgeons to acquire and master TAVR explantation techniques.

Performing TAVR explantation and AVR through a right minithoracotomy using a 3-dimensional totally endoscopic approach is highly advanced, and we believe this is the first documented case. Given the patient's advanced age of 85 years, a conventional full sternotomy would likely have led to a longer recovery time. However, minithoracotomy significantly reduced ventilator dependency and recovery time, leading to a favorable outcome.

Minimally invasive cardiac surgery offers several advantages, including reduced blood loss, a lower infection risk, decreased postoperative pain, faster recovery, a quicker return to daily activities, and smaller scars. However, it also presents greater technical challenges, necessitating a steep learning curve and carrying higher risks of neurological events, aortic dissection, and groin complications.^7^ While minimally invasive mitral valve surgery has advanced significantly, minimally invasive aortic valve surgery remains uncommon. Simultaneous TAVR explantation and AVR via minimally invasive techniques is even rarer. TAVR explantation is typically conducted via sternotomy because of the adhesions surrounding the TAVR prosthesis. However, the lasso technique enables easier TAVR explantation through a right minithoracotomy using a 3-dimensional totally endoscopic approach.

In our previously reported lasso technique performed during sternotomy, a 2-0 polyester purse-string suture was placed along the edge of the frame and secured with a tourniquet as a valve holder. However, in minimally invasive procedures, the tourniquet obstructs visibility and limits instrument movement, so the suture was tied directly.

In 2019, Hernandez-Vaquero et al^8^ introduced spatula endarterectomy during TAVR explantation; however, it is not suitable for minimally invasive surgery and poses a risk of tissue damage. In contrast, the lasso technique is easily adaptable to a minimally invasive setting, and the 3-dimensional totally endoscopic approach provides optimal visualization of the TAVR prosthesis and adjacent structures, facilitating both explantation and AVR. Although there have been advancements in attempts to perform AVR through right minithoracotomy, no reports exist of simultaneous TAVR explantation and AVR in elderly, high-risk patients. Therefore, this report represents a significant advancement in surgical techniques.

## Potential Pitfalls

The lasso technique simplifies TAVR explantation, but inadequate understanding of the prosthesis and surgical plane can cause iatrogenic injury to the sinotubular junction, aorta, mitral valve, or membranous septum. Therefore, gentle endarterectomy and traction are essential.

In addition, it is essential for not only surgeons but also interventionalists and imaging cardiologists to understand that TAVR explantation does not always need to be a highly complex procedure. Recognizing this can prevent instances in which patients with complications are referred for SAVR, often regretting their initial choice of TAVR. Timely referrals facilitate effective reoperation, and a thorough understanding of the TAVR prosthesis and surgical anatomy contributes to safer and more efficient explantation.

When the complexity of TAVR explantation is minimized, TAVR becomes an even more viable option. Furthermore, smaller incisions can be used instead of a full sternotomy, providing further benefits to the patient.

## Conclusions

We present a case of successful TAVR explantation and concomitant AVR using the lasso technique with a 3-dimensional, totally endoscopic, 2-port system approach. The lasso technique is applicable not only in sternotomy but also in minimally invasive cardiac surgery, offering a reproducible method with significant potential benefits.

## Funding Support and Author Disclosures

The study was approved by the Institutional Review Board of Asan Medical Center, which waived the need for informed consent (approval no: 2024-1337; date of approval: 2024-11-15). The authors have reported that they have no relationships relevant to the contents of this paper to disclose.

## Appendix

Video 1 Operative Videos of the Totally Endoscopic Approach for Transcatheter Aortic Valve Replacement Explantation Followed by Surgical Aortic Valve Replacement Using a 3-Dimentional Endoscope System

## References

[1] Mangner N., del Val D., Abdel-Wahab M. (2022). Surgical treatment of patients with infective endocarditis after transcatheter aortic valve implantation. J Am Coll Cardiol.

[2] Tang G.H.L., Zaid S., Kleiman N.S. (2023). Explant vs redo-TAVR after transcatheter valve failure: mid-term outcomes from the EXPLANTORREDO-TAVR international Registry. JACC Cardiovasc Interv.

[3] Bowdish M.E., Habib R.H., Kaneko T. (2024). Cardiac surgery after transcatheter aortic valve replacement: trends and outcomes. Ann Thorac Surg.

[4] Kim Y.S., Yoo J.S. (2022). Easy surgical explantation technique for self-expanding transcatheter aortic valve: 'lasso technique'. Interact Cardiovasc Thorac Surg.

[5] Carroll J.D., Mack M.J., Vemulapalli S. (2021). STS-ACC TVT Registry of transcatheter aortic valve replacement. Ann Thorac Surg.

[6] Yokoyama Y., Kuno T., Zaid S. (2021). Surgical explantation of transcatheter aortic bioprosthesis: a systematic review and meta-analysis. JTCVS Open.

[7] Falk V., Cheng D.C., Martin J. (2011). Minimally invasive versus open mitral valve surgery: a consensus statement of the international society of minimally invasive coronary surgery (ISMICS) 2010. Innovations (Phila).

[8] Hernandez-Vaquero D., Pascual I., Diaz R., Avanzas P., Moris C., Silva J. (2019). Surgical explantation of a transcatheter-implanted aortic valve prosthesis is feasible and easy. Ann Thorac Surg.

